# Tailor-Making a Protein A-Derived Domain for Efficient Site-Specific Photocoupling to Fc of Mouse IgG_1_

**DOI:** 10.1371/journal.pone.0056597

**Published:** 2013-02-12

**Authors:** Feifan Yu, Peter Järver, Per-Åke Nygren

**Affiliations:** Division of Molecular Biotechnology, Royal Institute of Technology (KTH), AlbaNova University Center, Stockholm, Sweden; MRC National Institute for Medical Research, United Kingdom

## Abstract

Affinity proteins binding to antibody constant regions have proved to be invaluable tools in biotechnology. Here, protein engineering was used to expand the repertoire of available immunoglobulin binding proteins via improvement of the binding strength between the widely used staphylococcal protein A-derived Z domain and the important immunoglobulin isotype mouse IgG_1_ (mIgG_1_). Addressing seven positions in the 58-residue three-helix bundle Z domain by single or double amino acid substitutions, a total of 170 variants were individually constructed, produced in *E. coli* and tested for binding to a set of mouse IgG_1_ monoclonal antibodies (mAbs). The best variant, denoted Z_F5I_ corresponding to a Phe to Ile substitution at position 5, showed a typical ten-fold higher affinity than the wild-type as determined by biosensor technology. Eight amino acid positions in the Z_F5I_ variant were separately mutated to cysteine for incorporation of a photoactivable maleimide-benzophenone (MBP) group as a probe for site-specific photoconjugation to Fc of mIgG_1_, The best photocoupling efficiency to mIgG_1_ Fc was seen when the MBP group was coupled to Cys at position 32, resulting in adduct formation to more than 60% of all heavy chains, with no observable non-selective conjugation to the light chains. A similar coupling yield was obtained for a panel of 19 different mIgG_1_ mAbs, indicating a general characteristic. To exemplify functionalization of a mIgG_1_ antibody via site-specific biotinylation, the Z_F5I-Q32C-MBP_ protein was first biotinylated using an amine reactive reagent and subsequently photoconjugated to an anti-human interferon-gamma mIgG_1_ mAb. When comparing the specific antigen binding ability of the probe-biotinylated mAb to that of the directly biotinylated mAb, a significantly higher bioactivity was observed for the sample biotinylated using the Z_F5I-Q32C-MBP_ probe. This result indicates that the use of a site-specific and affinity probe-mediated conjugation strategy can result in antibody reagents with increased assay sensitivity.

## Introduction

Antibodies play crucial roles in *in vitro* diagnostic assays of different complexity, involving either the use of single antibody reagents, or more commonly, by combining several antibodies in "sandwich" assays or more sophisticated set-ups [1]. In most cases, at least one of the antibodies used in the assay needs to be labeled allowing for direct or indirect detection via fluorescence, enzymatic conversion of suitable substrates, or DNA amplification/replication [2]–[4]. Such labeling of native antibody proteins is typically performed in a more or less uncontrolled manner in respect to the number and location of sites being modified. The approach typically employs N-hydroxysuccinimide (NHS) or maleimide-activated probes that address primary amines or free thiol groups, respectively, present in the antibody protein. The use of such labeling methods could potentially affect antigen binding activity, contribute to an increased cross-reactivity and complicate the use of reagents in quantitative assays in which information of the number of labels per antibody is important [5].

Recently, alternative means for obtaining a more controlled covalent labeling of antibodies have been described involving the recruitment of selective probes based on engineered and functionalized domains of naturally occuring bacterial Ig binding proteins, such as staphylococcal protein A (SPA) and streptococcal protein G (SPG). SPA or SPG domain variants have been designed to contain non-native groups capable of mediating covalent conjugation of the probe to the antibody protein after binding, either via photoactivation of the introduced group or via its high inherent chemical reactivity (e.g. electrophilic attack) [6]–[8]. If the Ig-binding probe also carries a reporter group, e.g. biotin, the end-result is a site-specifically labeled antibody ready for use in combination with e.g. streptavidin-based fluorescent conjugates. Similarly, the use of photoactivable Ig-binding probes for obtaining directed, covalent surface immobilization of antibodies has been described [7].

Thus, these approaches rely on the ability of a recruited bacterial Ig-binding domain to efficiently and selectively interact with a defined site in an antibody and a properly positioned functional group that is engaged in the formation of a covalent cross-link between the probe and an acceptor group in the antibody protein. The optimal positioning of the functional group is not entirely straightforward as the Ig-binding protein should be able to accommodate it without being significantly structurally destabilized. Further, a balance must be found between placement of the probe close to the protein-protein interaction interface, thereby gaining access to the antibody protein partner, but not so close as to negatively interfere with the binding in the first place. In addition, depending on the functional group used, e.g. benzophenone (BP) for photoinduced coupling or bis-acrylamide for electrophilic attack-based coupling, suitable acceptor sites in the antibody must be within reach to form the complex. Also, the potential for self-reactivity of the label, i.e. via internal cross-linking to sites within the probe itself, should be avoided.

For probe-based labeling technology to be of general applicability there is a prerequisite for the existence of suitable Ig-binding domains capable of binding to the particular Ig class or subclass of interest whilst also being amenable to the required engineering that involves site-specific introduction of functional groups for both the covalent coupling and subsequent reporting. Examples of useful functionalized probes include reagents based on the 58-residue SPA-derived three-helix bundle Z ("Z domain") that binds to the CH2-CH3 region in the "Fragment crystallizable" (Fc) of IgGs from different sources [9]. Z domain-based probes have proven to be amenable to production by both chemical derivatization after recombinant expression or via direct solid phase peptide synthesis and thus constitute an attractive framework for further development in this field.

However, a significant limitation associated with the Z domain is its relatively weak interaction with Fc of mouse IgG_1_ (mIgG_1_), that represents the prime isotype of murine monoclonal antibodies produced by hybridoma technology and widely used in *in vitro* diagnostics [10], [11]. In order to expand the applicability of Z domain-based probes for covalent labeling of immunoglobulins, we have investigated whether protein engineering can be used to increase the interaction strength between the Z domain and Fc of mIgG_1_. From comparison of amino acid sequence data from Z domain-binding and non-binding Ig isotypes, as well as 3D structural information, several candidate positions in the Z domain were identified and subjected to amino acid substitution. The resulting proteins were tested for binding to mIgG_1_ and a preferred variant showing a significantly increased affinity to mIgG_1_ was identified. An ideal position for site-specific incorporation of a photoreactive BP group for coupling to mIgG_1_ was found via scanning mutagenesis, resulting in a final reagent that shows efficient photoinduced covalent cross-linking to a large panel of monoclonal mIgG_1_ reagents. Furthermore, successful use of the probe for Fc-specific biotinylation of a mouse IgG_1_ mAb was demonstrated. These results suggest that this overall strategy can yield reagents of higher antigen binding activity compared to direct mAb biotinylation.

## Results

### Mutagenesis and binding analyses of novel Z domain variants

**Figure 1:**
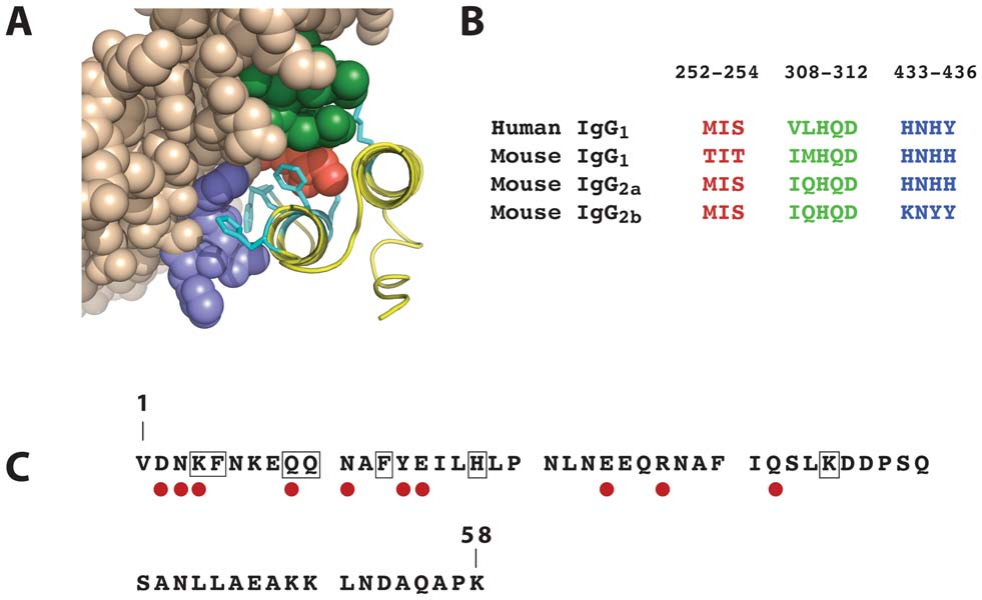
Structure and sequence data. (**A**): Computer graphic representation of a part of the complex between a single staphylococcal protein A domain (domain B, closely related to the Z domain used in the present study) (yellow) and human IgG_1_ Fc (brown) (PDB file: 1FC2.pdb). The amino acid side chains corresponding to the seven positions addressed for substitutions in the Z domain are highlighted in cyan. Highlighted in purple, red and green, respectively, are three IgG1 Fc subregions in close contact with the B domain. (**B**): Alignment of amino acid sequences of Fc regions of human IgG_1_, mouse IgG_1_, mouse IgG_2a_ and mouse IgG_2b_, respectively, corresponding to the three contact areas shown in (A), using the same colour code. (**C**): Amino acid sequence of the 58-residue Z domain, with the seven positions included in the engineering boxed. Indicated with red dots are the ten positions at which unique cysteine residues were introduced for site-specific labeling with a photoactivable maleimide benzophenone (MBP) group.

**Figure 2:**
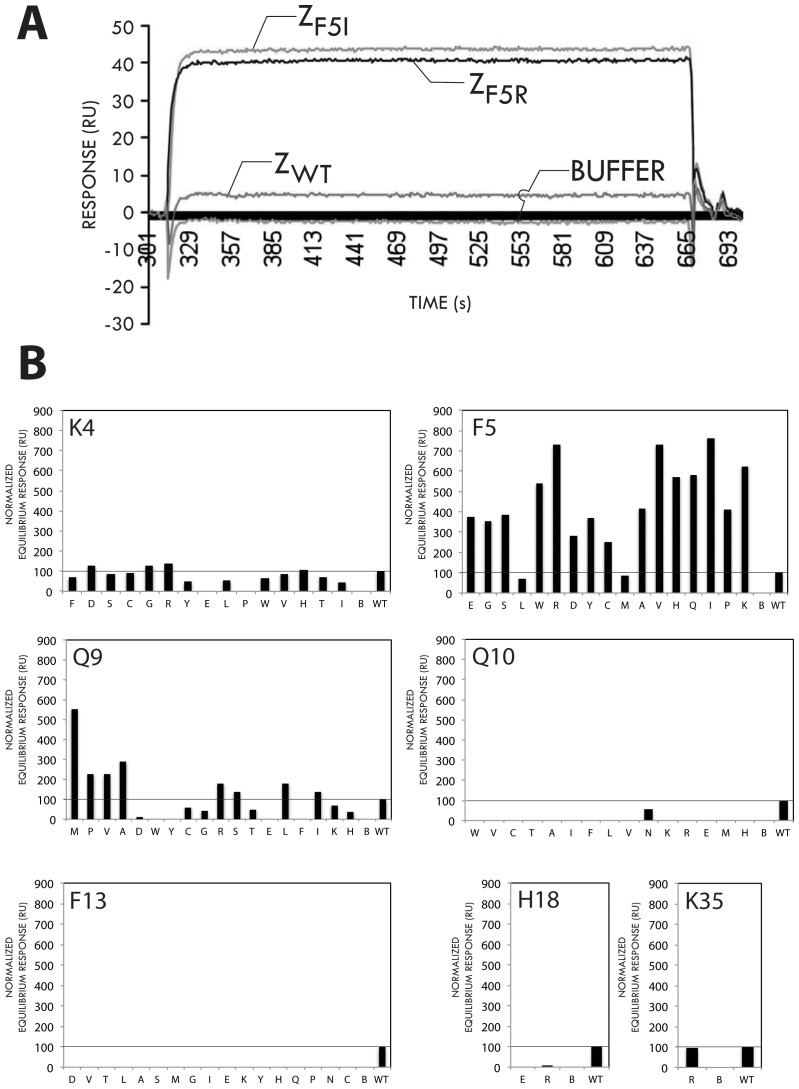
Relative binding responses for Z domain variants to mouse IgG_1_. Solutions of Z domain variants (2 µM concentration) obtained after single amino acid substitutions at the seven targeted positions (K4, F5, Q9, Q10, F13, H18 and K35) were injected over a sensor chip surface containing immobilized mIgG_1_ monoclonal antibody protein and the equilibrium responses (plateau values) were recorded and normalized to the response values obtained for the wild type Z domain (WT) A blank surface (activated/deactivated) was used as negative control and used for buffer effect subtraction. (**A**) A representative overlay sensorgram from injections of Z_WT_, Z_F5R_ and Z_F5I_ variants over mIgG_1_ monoclonal protein showing higher equilibrium response values for the two mutant variants than for the Z_WT_ domain. The response obtained from buffer injection only is also indicated (BUFFER). (**B**) Results from the analysis of the different variants. The horizontal line in each panel corresponds to the normalized response obtained for the wild type Z domain ( = 100).

In an attempt to identify variants of the wild type Z domain (Z_WT_) showing increased affinity for Fc of mouse IgG_1_ (mIgG_1_), a number of mutants containing either single or double amino acid substitutions were designed, produced and tested for binding to different mIgG_1_ monoclonal antibodies (mAbs). The selection of positions in the 58-residue domain for mutagenesis was based on analysis of both available 3D structural data and aligned amino acid sequences of different human and mouse Fc regions that have been reported to exhibit different affinity for SPA, from which the Z_WT_ domain originates. A three-dimensional structure of the complex between an individual Protein A domain (domain B, the protein ancestor of Z_WT_) and Fc of human IgG_1_ suggests that three main Fc regions contact the SPA domain [12] (Figure 1A). Within these regions, there are amino acid sequence differences between human IgG_1_ Fc (strong Z_WT_ interaction) and murine IgG_1_ (non/weak Z_WT_ interaction). From analysis of the hIgG_1_/domain B complex, including data from an earlier NMR-based study of the same complex [13], seven residues in the Z_WT_ domain that point into regions of hIgG_1_/mIgG_1_ sequence variation were selected for mutagenesis. In the first set of constructs these positions were individually addressed and, using degenerate codons, several variants corresponding to each position were constructed simultaneously as a mixed pool, for later clonal separation and identification via DNA sequencing. To this end, positions K4, F5, Q9, Q10 and F13 were all separately mutated using (N)(N)(G/T) degenerate codons, to theoretically yield all 19 non-native variants for each position. However, in the subsequent cloning and identification work, typically involving the sequencing of 96 clones per position, one or two of the expected variants were not found at any given position and are therefore not included in the study. Positions H18 and K35 were more narrowly mutated, to yield H/E/R and K/R variants, respectively. This first set of 100 Z domain variants, was expressed in separate *E. coli* cultures as N-terminal hexahistidyl (His_6_) fusions and purified by IMAC, prior to binding analyses against different Ig reagents performed using surface plasmon resonance In the initial screen, all variants were separately injected as analyte (2 µM concentration) over sensor chip surfaces harboring three different immobilized mIgG_1_ monoclonal antibodies. The equilibrium response signals were compared to that obtained for the Z_WT_ domain. For the majority of 41 investigated variants corresponding to substitutions at positions 10, 13, 18 and 35, the results showed either lower or only marginally increased binding responses to the mouse IgG_1_ mAb panel (Figure 2, showing data for one mIgG_1_ mAb). Of 18 position 9 variants, only four variants showed an increased binding response, of which the variant a Z_Q9M_ performed best. For position 4 variants, a few of the introduced substitutions resulted in only marginally higher relative binding responses, as seen for variants denoted Z_K4R_, Z_K4D_ and Z_K4G_. The largest effects were seen for substitutions at position 5, for which 16 out of 18 tested mutants showed higher binding responses than for the Z_WT_ domain. Of these, the variants Z_F5R_ and Z_F5I_ showed the highest binding responses to all three mIgG_1_ target proteins tested. In a separate experiment, the dissociation constants (K_D_) for 1∶1 interactions between Z_F5R_ and the Z_F5I_ variants and a representative mIgG_1_ mAb were determined to be approximately 10 µM (using equilibrium response values due to the fast binding and dissociation kinetics). This value should be compared with a corresponding K_D_>100 µM observed for the Z_WT_ domain, and thus indicates that these particular single amino acid substitutions result in more than ten-fold higher affinity for mIgG_1_ Fc.

**Figure 3:**
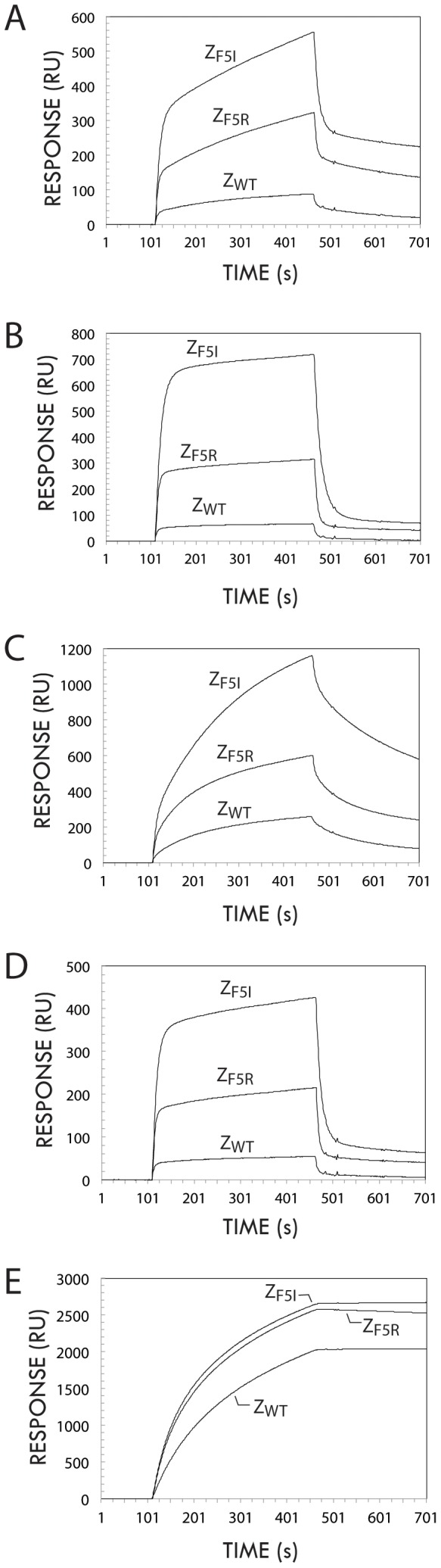
Binding of antibodies to immobilized Z domain variants. (**A-D**): Overlay of sensorgram traces obtained after injection of four different mouse IgG_1_ monoclonal antibodies (one per panel) over separate sensor chip surfaces containing similar amounts of the variants Z_F5I_, Z_F5R_ or the wild type Z domain (Z_WT_), respectively. (E): Overlay of sensorgram traces obtained after injection of a TNF-alpha receptor:human IgG1 Fc fusion protein (etanercept, Enbrel®) over separate sensor chip surfaces containing similar amounts of the variants Z_F5I_, Z_F5R_ or the wild type Z domain (Z_WT_), respectively.

The binding characteristics of the Z_F5R_ and the Z_F5I_ variants were further characterized using a reverse set-up, involving the injection of different mIgG1 mAbs over separate sensor chip surfaces harboring similar amounts of immobilized Z_F5R_, Z_F5I_ or Z_WT_ ligands (1092, 1163 and 1422 RUs, respectively). The increased binding strength to different mIgG1 mAbs for the two variants was evident, with the highest binding responses seen for the Z_F5I_ variant for all three mIgG_1_ mAb analytes (Figure 3A-C). A comparison of the sensorgram profiles obtained using the two different formats, i.e. using the Z variants either as injected analytes or as immobilized ligands, suggests that avidity effects resulting in slower dissociation kinetics are present in the latter case, as could be expected from the symmetric homodimeric nature of Fc proteins.

Further, the Z domain binding to human IgG_1_ Fc, in the form of a recombinant hTNF-alpha receptor-hIgG1 Fc fusion (etanercept, Enbrel®) as analyte, was also tested to investigate whether the substitutions affect the strong binding displayed by Z_WT_ to this immunoglobulin class. Interestingly, both the Z_F5R_ and the Z_F5I_ ligands bound to hIgG_1_ Fc with similar, or even slightly increased efficiency, compared to the Z_WT_ ligand (Figure 3D).

Based on the positive effects seen for single position substitutions at positions 5, 9 and to some extent position 4, it was decided to construct a limited number of double substitution variants to investigate whether cooperative effects could be obtained. To this end, (N)(N)(G/T) degenerate codons were separately introduced both at positions 4 and 9 in Z_F5I_ and Z_F5R_ template variants, respectively, to simultaneously construct all gene variants in pools for later individual identification via DNA sequencing. Hence, four new subsets of variants including in total 70 variants (six missed variants from the 76 possible variants) were constructed, sequenced and individually expressed. The results, however, showed that none of this class of variants bound more strongly than the highest affinity single substitution variants (data not shown).

### Site-specific incorporation of the photoactivable probe benzophenone

**Figure 4:**
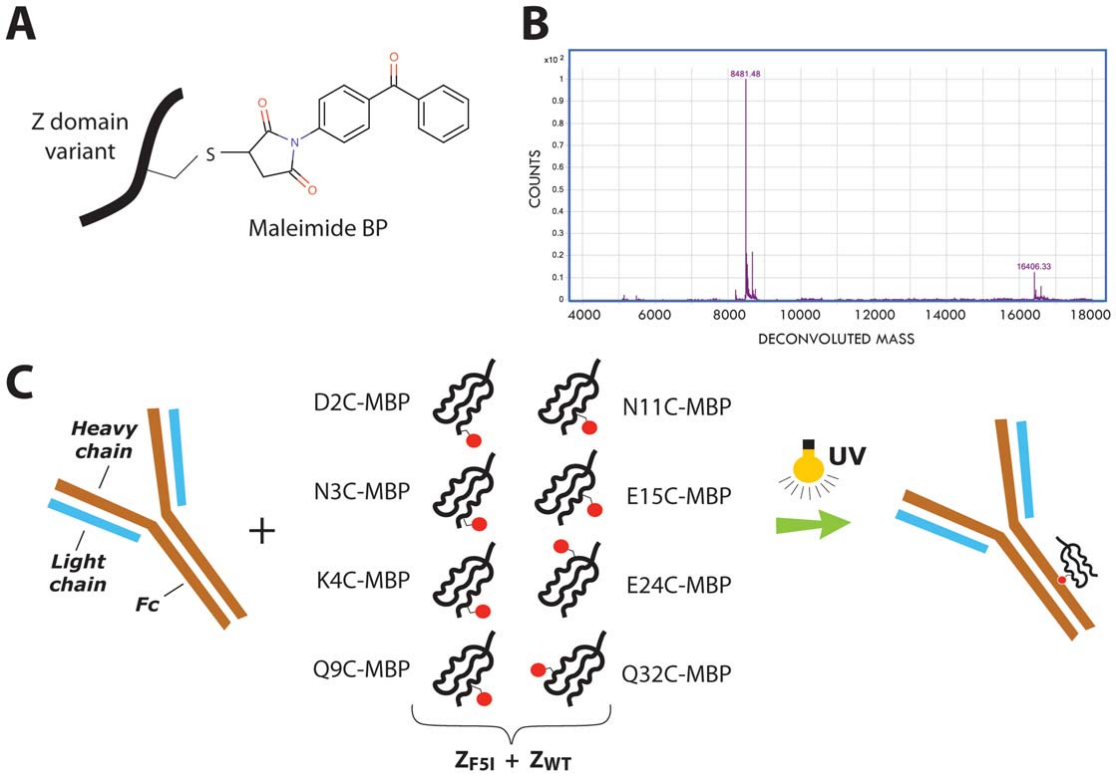
Site-specific conjugation of maleimide benzophenone to Z domain variants. (**A**): Schematic showing how maleimide benzophenone is conjugated to a unique cysteine residue introduced into each of the Z variants by genetic engineering. (**B**): A representative result from analysis by LC-ESI mass spectrometry of the product obtained after conjugation (Z_F5I-Q32C-MBP_). (**C**): Schematic showing the 16 different Z-MBP probes (eight positions for the MBP group on both the Z_WT_ and Z_F5I_ variants) evaluated for photocoupling to Fc of mIgG_1_ using UV radiation.

The Z_F5I_ variant was used as template for incorporation of a benzophenone (BP) probe for mediating photoinducable covalent cross-linking to mIgG_1_ Fc. Since the optimal positioning of the probe depends on several parameters that are difficult to model, ten separate candidate positions were investigated in parallel. To obtain site-specific incorporation, the wild type amino acid at each site was genetically exchanged for a unique cysteine residue, followed by incorporation of the BP group using a thiol-reactive maleimide-BP reagent (MBP). The ten positions in the evaluation were D2, N3, K4, Q9, N11, Y14, E15, E24, R27 and Q32 (Figure 1C). For comparison in later coupling studies, a corresponding set of ten variants of the original Z_WT_ domain was also procured by the same procedure. For unknown reasons two of the variants, Y14C and R27C, were difficult to express and conjugate, as both Z_WT_ and Z_F5I_ versions, and could not be assessed further. Analysis of the remaining eight conjugates by ESI-MS showed a high efficiency of MBP incorporation and the correct molecular weight (Figure 4).

### Photoactivated covalent coupling of probes to mIgG_1_ Fc

**Figure 5:**
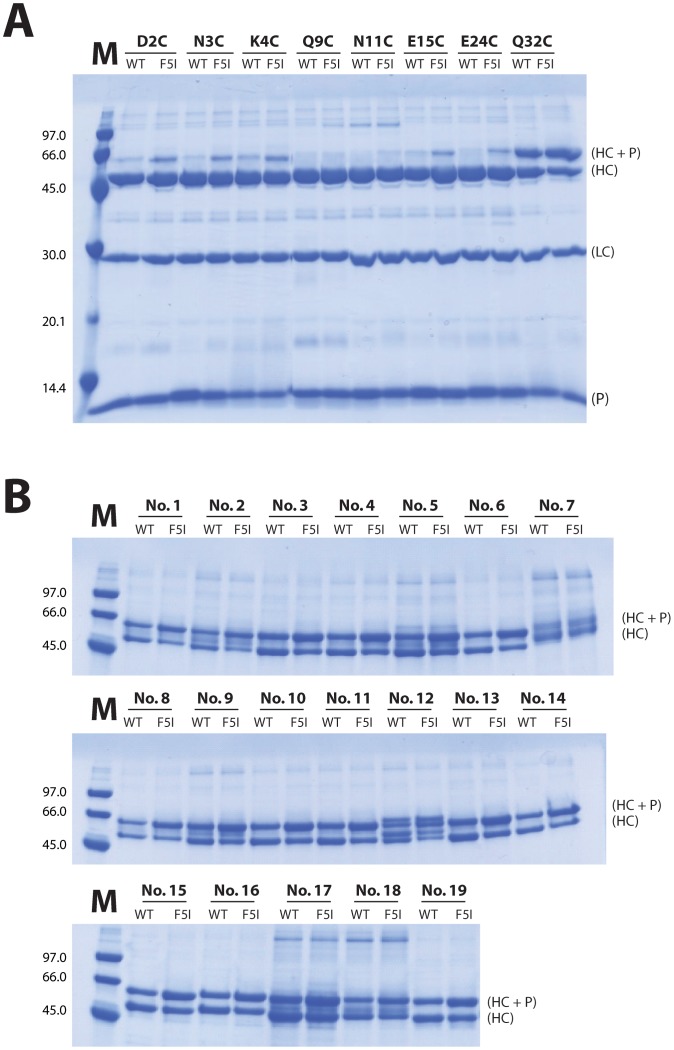
Photo-conjugation of Z domain probes to mouse IgG_1_. (**A**): Analysis by SDS-PAGE of the mouse IgG_1_ mAb photocoupling efficiency of nine different Z_WT_- or Z_F5I_-based probes, differing in the position of the maleimide benzophenone (MBP) group. As indicated, variants containing the MBP group at positions 2, 3, 4, 9, 11, 15, 24 or 32 were investigated. (**B**): Analysis by SDS-PAGE of the photocoupling efficiency of the Z_WT-Q32C-MBP_ and Z_F5I-Q32C-MBP_ probes, both containing the MBP group at position 32, to 19 different mouse IgG_1_ mAbs (see Material and Methods for a list). The designations HC+P, HC, LC and P, refer to heavy chain+probe, heavy chain, light chain and probe, respectively. The M lanes refer to marker protein with molecular weights in kDa as indicated.

**Figure 6:**
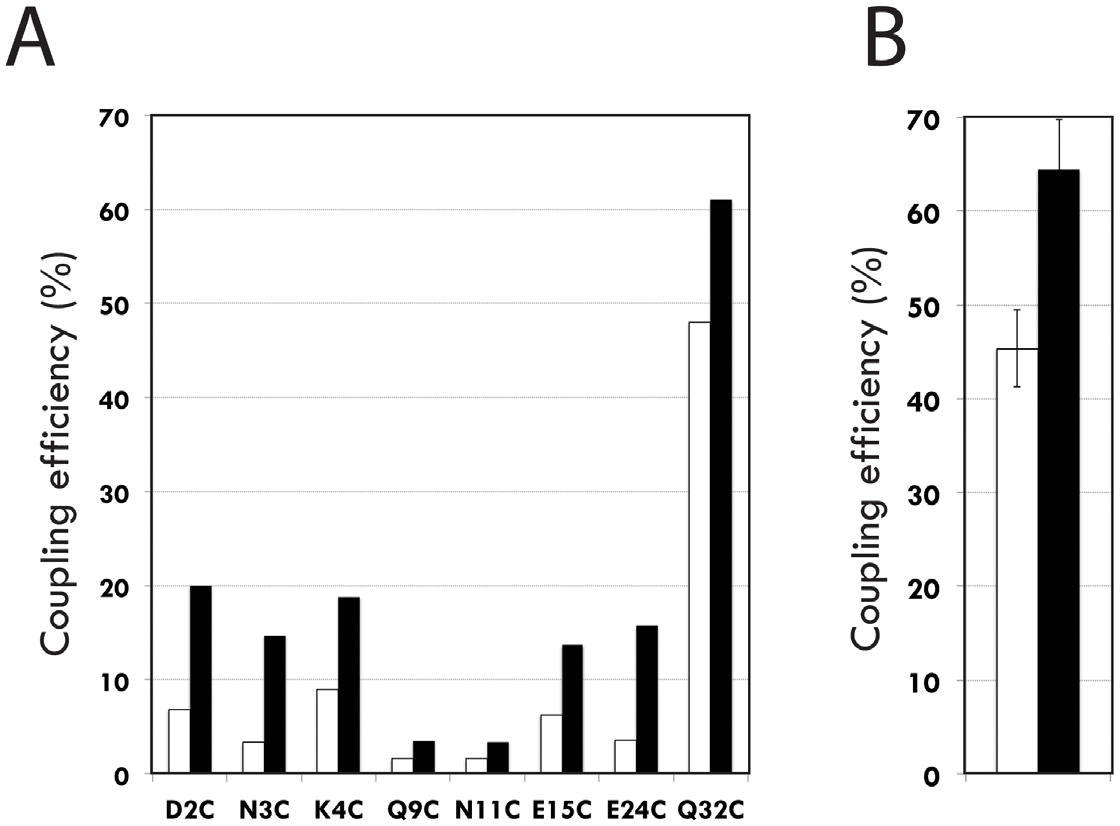
Photo-conjugation efficiencies for the different Z domain probes to mouse IgG_1_. The efficiencies of photoconjugation were analysed through scanning of the gel images in Figure 5 using ImageJ software (see Materials and methods section). (**A**) Photo-conjugation efficiencies for 16 probe variants, derived from either the Z_WT_-domain (white bars) or the Z_F5I_-domain, containing the F5I substitution (black bars). The position at which a cysteine was introduced to site-specifically position the MBP probe is indicated for each probe. (**B**) Mean values of photo-conjugation efficiencies (%) from the use of either the Z_WT-Q32C-MBP_ (white bar; mean value  =  45.4±4.1%) or Z_F5I-Q32C-MBP_ (black bar; mean value  =  64.4±5.3%) probes to a large panel of different mIgG_1_ antibodies (16 of the mAbs seen in Figure 5B).

In an initial series of experiments, a single mIgG_1_ monoclonal mAb (HDL 110) was used as acceptor target and the products of the coupling process were analyzed by SDS-PAGE under reducing conditions. The coupling efficiency was monitored through a comparison of the relative amount of heavy chain either carrying, or not carrying, an extra 8 kDa mass as a result of the covalently attached Z domain derivative. The results showed that the coupling efficiency varied considerably depending on both the position of the MBP group and on the presence or not of the affinity-enhancing F5I substitution in the Z-MBP probes (Figures 5A and 6A). For two positions of the MBP group, corresponding to coupling at Q9C and N11C, only very low levels of photoaddition of the probe to mIgG_1_ Fc could be observed, even for the Z domain variant containing the F5I substitution. In Z domain probes where the MBP group was positioned at D2C, N3C, K4C, E15C or E24C, photoaddition efficiencies of 13-20% were typically seen for the Z-variants containing the F5I substitution, whereas the levels for the corresponding Z_WT_-derived variants were significantly lower (3-9%), indicating a beneficial effect from the F5I substitution. Interestingly, the two probes having the MBP group coupled to the Q32C position showed significantly higher photoaddition efficiencies, with levels of Fc coupling of 48% and 61% for the Z_WT_ and Z_F5I_ variants, respectively. As before, the higher coupling efficiency was observed for the probe harbouring the affinity enhancing F5I substitution. No modification of the light chain was observed in any of the samples, indicating that the photoaddition reaction is selective for the protein to which the Z domain protein binds by "true" biomolecular recognition. In addition, no dimers or multimers of the Z-domain probe itself were observed, again indicating a degree of selectivity in the photoinduced coupling that depends on molecular recognition.

To investigate whether the observed coupling of the Z_F5I-Q32C-MBP_ probe to mIgG_1_ Fc proteins is a general property, a panel comprising 18 additional mIgG_1_ mAbs was used in a second series of photoaddition experiments. The results showed that for the mIgG_1_ tested high coupling efficiencies were generally obtained, again selectively targeted to the heavy chains (Figure 5B). In all cases, use of the Z_F5I-Q32C-MBP_ probe showed higher coupling efficiencies (mean value of 64.4±5.3%, based on 16 mAbs) than the Z_WT-Q32C-MBP_ probe (mean value of 45.4±4.1%), supporting the notion of a beneficial effect from an increased affinity for the mIgG_1_ Fc target (Figure 6B).

### Fc-specific biotinylation of a mouse monoclonal antibody

**Figure 7:**
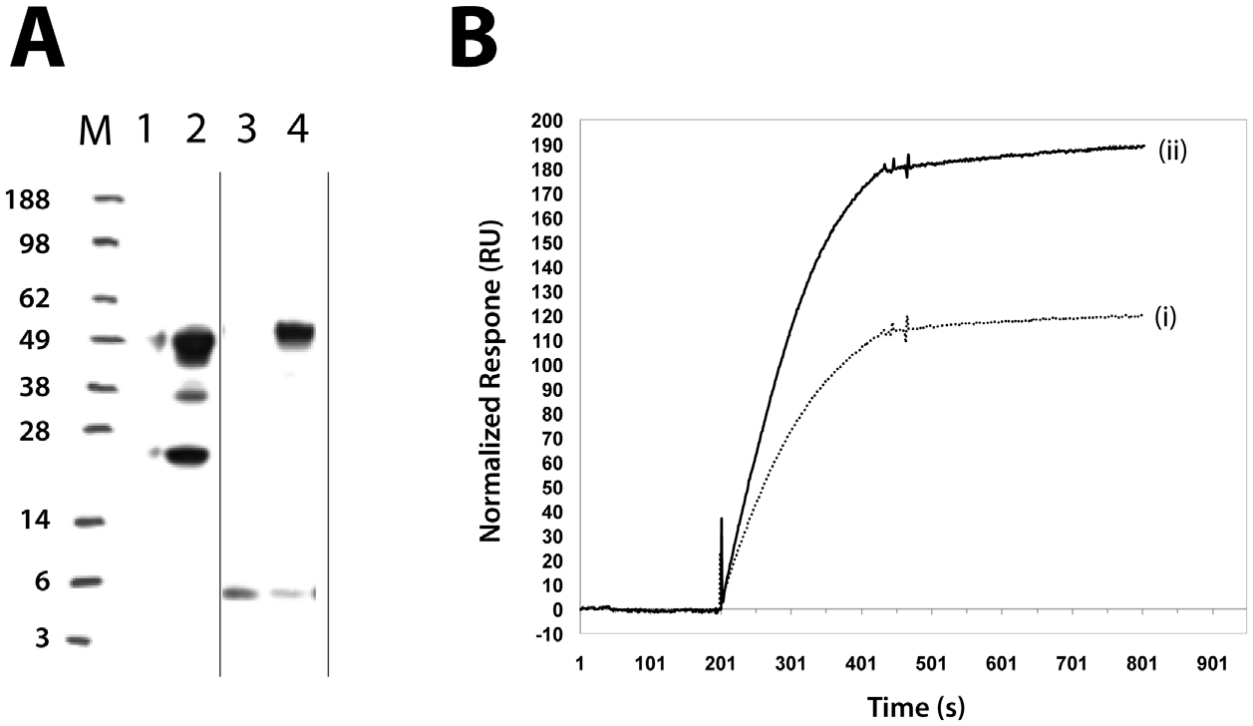
Analysis of labeling and comparison of the antigen binding potency of differently biotinylated antibody samples. Two samples of a mouse anti-human interferon-gamma mIgG1 mAb were biotinylated using either a direct or a Z_F5I-Q32C-MBP-BIO_ probe-mediated strategy and the resulting preparations compared with respect for their antigen binding potency. (**A**) Western blotting analysis of the samples and the probe, using a streptavidin horse radish peroxidase (HRP) conjugate. Lane 1: blank; lane 2: a sample of the anti-human interferon-gamma mIgG1 mAb biotinylated using a conventional amine reactive sulfo-NHS-ester-biotin reagent resulting in "global" biotinylation of both heavy (*ca.* 50 kDa) and light chains (*ca.* 25 kDa); lane 3: the Z_F5I-Q32C-MBP-BIO_ probe alone, biotinylated using the same amine reactive sulfo-NHS-ester-biotin reagent; lane 4: a sample of the anti-human interferon-gamma mIgG1 mAb after photoconjugation to the Z_F5I-Q32C-MBP-BIO_ probe (ca. 8 kDa) showing a selective biotinylation of only the heavy chains (*ca.* 50 + 8 kDa). A small amount of unreacted probe is also visible. (**B**) Biosensor analysis of the relative antigen binding potency of the biotinylated antibody preparations. Using a streptavidin coated biosensor chip, the biotinylated antibody proteins were selectively immobilized onto separate sensor chip surfaces, followed by injection over both surfaces of a common 7.5 nM solution of the antigen human interferon-gamma. This allowed for direct comparison of the effect on the antigen binding potency from the different biotinylation strategies. Sample (i): the anti-human interferon-gamma mIgG_1_ mAb biotinylated using a conventional amine reactive sulfo-NHS-ester-biotin reagent; sample (ii): the anti-human interferon-gamma mIgG_1_ mAb biotinylated via photoconjugation using the Z_F5I-Q32C-MBP-BIO_ probe.

To investigate the use of the Z_F5I-Q32C-MBP_ conjugate for antibody functionalization, the reagent was employed for biotinylation of a single mIgG_1_ mAb. The Z_F5I-Q32C-MBP_ probe was first biotinylated using a sulfo-NHS-ester-biotin reagent that targets free amino groups. The Z domain contains six lysines and the N-terminal alpha-amino group, so has the capacity for such coupling. Successful biotinylation was demonstrated by streptavidin-horse radish peroxidase (SA-HRP) conjugate-based Western blot analysis of a sample of the Z_F5I-Q32C-MBP_ probe (Figure 6A, lane 3). An aliquot of the biotinylated Z_F5I-Q32C-MBP_ probe was subsequently used for photocoupling to an anti-human interferon-gamma mIgG_1_ mAb. SA-HRP Western blotting showed that the procedure resulted in a selective biotinylation of the mAb heavy chain only; no staining could be detected for the light chain (Figure 6A, lane 4). For comparison, a sample of the same anti-human interferon-gamma mIgG_1_ mAb was also subjected to standard biotinylation, i.e. using the amino-reactive sulfo-NHS-ester-biotin reagent directly. As expected, Western blot analysis of this sample showed that both the light and heavy chains of the antibody were biotinylated (Figure 7A, lane 2). In addition, it was confirmed that biotinylation of the mAb using the bio-Z_F5I-Q32C-MBP_ probe was directed to the Fc fragment of the heavy chain (i.e. the CH_2_-CH_3_ domains), and not to the domains of the heavy chain that are part of the F(ab´)_2_ fragment of the antibody (i.e. the VH and CH_1_ domains) (see Supportive Information: Experiment S1).

A surface plasmon resonance study was performed to investigate whether the probe-mediated, Fc-specific biotinylation of the mAb confers any advantage over the standard undirected biotinylation, with respect to the reagent antigen-binding capacity. To this end, aliquots of the two biotinylated reagents were injected over separate streptavidin-coated sensor chip surfaces. For both chip surfaces, the injection of a 7.5 nM solution of the human interferon-gamma antigen resulted in a significant binding response. Interestingly, when the binding responses are normalized for the slightly different immobilization levels for the two reagents, it becomes evident that the antigen binding response per unit of immobilized antibody is 60% higher for the mAb sample conjugated with biotinylated Z_F5I-Q32C-MBP_ (Figure 7B). This result indicates that the Fc-specific derivitization strategy via the Z domain-MBP conjugate results in a biotinylated mAb reagent with superior specific activity.

## Discussion

The approach to investigate the effect of single amino acid substitutions to find Z domain variants with higher affinity for mouse IgG_1_ Fc was inspired by the earlier work of Nagaoka and Akaike [10] who used a similar approach to improve the affinity of this protein-protein interaction. However, this earlier study focused on the engineering of the Fc part of the complex. They identified that a single threonine to methionine substitution at position 252 in mIgG_1_ Fc increased the affinity to a practically useful range, and thereby showed that relatively small changes can significantly affect the interaction. In the present study, we have focused on engineering the Z domain because, even if the development of transgenic mice strains containing this engineered Fc-gamma allele could be envisioned, we reasoned that a Z domain-based reagent with improved affinity to pre-existing (and continuously developed) "wild type" mIgG_1_ monoclonal antibodies would be valuable.

The variant denoted Z_F5I_, that showed the highest affinity to a panel of mouse IgG_1_ mAbs tested, binds with a K_D_ of *ca.* 10 µM. Although this binding strength cannot be regarded as high, it nevertheless corresponds to at least a 10-fold increase and, as seen in the mAb conjugation experiments, contributes to an increased labeling yield. The lack of native cysteines in the Z domain allowed for cysteine-maleimide chemistry to be used for site-specific incorporation of the BP group into the protein. The most beneficial position (at residue Q32) for introduction of the photoactivable MBP group was found after empirical testing of eight candidate positions. The results suggest that, at least for this interaction, the BP group should ideally be positioned outside of the core interaction surface. As important, it should also be positioned so that it photoreactive centre can reach a suitable acceptor group in the Fc binding partner. However, it is presently not known with which group, or groups, the BP reacts during covalent attachment to Fc.

In this study the incorporation of the biotin group into the Z_F5I-Q32C-MBP_ probe was achieved via undirected amine coupling. However, alternatives to this strategy exist such as through recombinant production of the probe as a genetic fusion to a biotin acceptor, such as the biotin carboxyl carrier protein (BCCP) [14] or shorter peptides that mimic the natural substrate [15], [16], which would give better control of the number (one) and position of the introduced biotin. Further, the probe could also be produced via chemical peptide synthesis, during which one or more biotins could be introduced in a site-specific manner. A peptide synthesis production route could also be used to introduce a BP group at position 32 in the form of the commonly used non-natural amino acid benzophenone alanine (BPA). However, the photocoupling efficiency may be sensitive to even small differences in the distance between the reactive BP group and the acceptor site; in this respect the cysteine side chain plus maleimide group and alanine side chain "linker" groups, respectively, could differ in their ‘reach’.

The coupling yield using the Z_F5I-Q32C-MBP_ probe exceeded 50%, which must mean, that some of the instrinsically homodimeric Fc chains carried more than one Z_F5I-Q32C-MBP_ probe. The absence of dimers or multimers of the probe after the photocoupling, which would have be evident from molecular weights corresponding to multiples of 8 kDa, does not rule out that a certain degree of self-reactivity may have been present. However, in such an event, it appears to have occurred in *cis*, i.e. intramolecularly between the MBP probe and an acceptor group within the same Z domain molecule.

The biosensor-based antigen binding assay of the biotin-streptavidin immobilized antibody reagents showed that the samples biotinylated via the Z_F5I-Q32C-MBP_ probe displayed a higher "bioactivity". The reasons for the lower activity for the randomly biotinylated sample could be several, including possible biotinylation of lysines in the complementarity determining regions (CDRs) thus directly affecting residues involved in antigen binding, or in regions that lead to unproductive orientation of the antibody after streptavidin immobilization.

In conclusion, the Z_F5I_ domain variant identified by screening a substantial number of single amino acid substitutions showed a significantly increased affinity to the mIgG_1_ target, and via the site-specific introduction of a photoconjugation agent and position Q32, provides the basis for a useful reagent in site-specific biotinylation of antibodies.

## Materials and Methods

### Bacterial strains, enzymes

The *E. coli* strain Rosetta (DE3) (Novagen) was used for vector construction and protein expression. Phusion Polymerase (Finnzymes), T4 DNA ligase (New England Biolabs), *Nhe*I and *Xho*I restriction enzymes (New England Biolabs) were used according to the suppliers recommendations.

### Plasmid preparation and cassette mutagenesis

The plasmid pT7HisZ_WT_, based on the pET-21 series (Novagen), and containing the Z_WT_ gene was purified by QIAquick DNA purification kit (Qiagen), cut with *Nhe*I and *Xho*I restriction enzymes and gel-extracted using the JETQUICK gel extraction kit (Genomed). The site-direct mutagenesis was achieved via cassette mutagenesis where cassettes were produced via oligonucleotide extension reactions using Phusion DNA polymerase under the following conditions: 98°C for 3 minutes; 10 cycles of (98°C for 10 seconds, ramping from 98°C to 70°C with 0.1°C decrease per second), 70°C for 30 seconds, and a final period of 72°C for ca. 10 minutes. This was followed by cleavage with *Nhe*I and *Xho*I restriction enzymes and purification with a QIAquick DNA purification kit (Qiagen). The following oligonucleotide combinations were used: (K4_for + Zwt _Rev), (F5_for + Zwt _Rev), (Q9NNK + Zwt _Rev), (Q10_for + Zwt _Rev), (F13NNK + Zwt_Rev), (H18DE + Zwt_Rev), (H18RQ + Zwt_Rev), (K35_Rev + Z wt_For), (K4NNKF5RI + Zwt_Rev), and (Q9NNKF5RI + Zwt_Rev). Inserts and plasmids were ligated using T4 DNA ligase, incubated at room temperature for 2 hours and then transformed into chemically competent *E. coli* Rosetta (DE3) cells, which were placed on kanamycin (50 µg/ml, Sigma-Aldrich) and chloramphenicol (20 µg/ml, AppliChem) agar plates at 37°C overnight.

Variants Z_WT_ or Z_F5I_ with additional substitutions D2C and N3C were constructed using PCR with designed forward primers: 5′-CACTACTACCTCGAGGTATGCAACAAA-3′ and 5′- CACTACTACCTCGAGGTAGACTGCAAA-3′ for D2C and N3C, respectively, in comabination with the common reverse primer 5′-TTTTAGCTTCTGCTAGCAA-3′. Variants Z_WT_ or Z_F5I_ with additional K4C, Q9C, N11C, Y14C, E15C, E24C, R27C or Q32C substitutions were identified from screening individual clones resulting from a random mutagenesis using oligonucleotides containing NNK degenerate codons at the respective positions.

**Oligonucleotides (Eurofins MWG Operon) used for gene constructions were designed as follows: Q10_for: 5**′**-**CACTACTACCTCGAGGTAGACAACAAATTCAACAAAGAACAA**NNK**AACGCGTTCTATGAGATCTTACATTTACCTAACTTAAACGAAG-3′; Zwt_for: 5'- CACTACTACCTCGAGGTAGACAACAAATTCAACAAAGAACAA**CAA**AACGCGTTCTATGAGATCTTACATTTACCTAACTTAAACGAAG-3'; Zwt _Rev: 5'-TTAGCTTCTGCTAGCAAGTTAGCGCTTTGGCTTGGGTCATCTTTTAAACTTTGG ATGAAGGCGTTTCGTTGTTCTTCGTTTAAGTTAG-3'; F5_for: 5'-CACTACTACCTCGAGGTAGACAACAAA**NNK**AACAAAGAACAACAAAACGCGTTCTATGAGATCTTACATTTACCTAACTTAAACGAAG-3'; K4_for: 5'-CACTACTACCTCGAGGTAGACAAC**NNK**TTCAACAAAGAACAACAAAACGCGTTCTATGAGATCTTACATTTACCTAACTTAAACGAAG-3'; K35_Rev: 5'-TTAGCTTCTGCTAGCAAGTTAGCGCTTTGGCTTGGGTCATCACGTAAACTTTGGATGAAGGCGTTTCGTTGTTCTTCGTTTAAGTTAG-3'; F13NNK: 5’-CACTACTACCTCGAGGTAGACAACAAATTCAACAAAGAACAACAAAACGCG**NNK**TATGAGATCTTACATTTACCTAACTTAAACGAAG-3’; H18DE: 5’-CACTACTACCTCGAGGTAGACAACAAATTCAACAAAGAACAACAAAACGCGTTCTATGAGATCTTAGAMTTACCTAACTTAAACGAAG-3′; H18RQ: 5'- CACTACTACCTCGAGGTAGACAACAAATTCAACAAAGAACAACAAAACGCGTTCTATGAGATCTTACRGTTACCTAACTTAAACGAAG-3'; Q9NNK: 5'-CACTACTACCTCGAGGTAGACAACAAATTCAACAAAGAA**NNK**CAAAACGCGTTCTATGAGATCTTACATTTACCTAACTTAAACGAAG-3'; K4NNKF5RI: 5’-CACTACTACCTCGAGGTAGACAAC**NNK**AKAAACAAAGAACAACAAAACGCGTTCTATGAGATCTTACATTTACCTAACTTAAACGAAG-3′; Q9NNKF5RI: 5′-CACTACTACCTCGAGGTAGACAACAAAA**K**AAACAAAGAA**NNK**CAAAACGCGTTCTATGAGATCTTACATTTACCTAACTTAAACGAAG-3′.

### DNA sequencing

Colonies appearing on plates were randomly picked, and plasmid inserts identified by DNA sequencing on an ABI Prism 3700 analyzer (Applied Biosystems). The primer pair LaMa 27: 5′-ATTAATACGACTCAC-3′ and WiMa32: 5′-ACACCCGCCGCGCTTAATGC-3′ were used to for colony PCR and sequencing reactions. Big Dye terminators (Amersham Biosciences) were used for BigDye Thermo Cycle Sequencing PCR. Sequencing PCR reactions were ethanol precipitated.

### Protein production and purification

Colonies were inoculated in TSB+Y (30 g/l tryptic soy broth, 5 g/l yeast extract, Merck, Germany) medium containing kanamycin (50 µg/ml) and chloramphenicol (20 µg/ml) at 37°C overnight. Cultures were diluted in 1∶1000 in 100 ml TSB+Y medium containing kanamycin (50 µg/ml) and chloramphenicol (20 µg/ml) in a 1-liter flask and cultured at 37°C until the OD_600 nm_ reached ca. 0.8–1.0. Subsequently, isopropyl ß-D-thiogalactoside (IPTG; Apollo Scientific Ltd, 0.5 M) was added to a final concentration of 1 mM to induce protein production, and the temperature lowered from 37 to 25°C. After overnight incubation, the cells were harvested by centrifuging at 2400 *g*, at 4°C for 8 minutes and subsequently cell pellets were suspended in 5 ml denaturing buffer (6 M guanidinium hydrochloride, 47 mM Na_2_HPO_4_, 2.65 mM NaH_2_PO_4_, 10 mMTris-HCl, 104 mM NaCl, pH 8.0) and shaken at 37°C for 2 hours. The suspensions were centrifuged at 35 000 *g*, at 4°C for 20 minutes and the cell lysates filtered (0.45 µm). The hexahistidyl-tagged proteins were purified using immobilized metal ion affinity chromatography (IMAC) using Talon Metal Affinity Resin (BD Biosciences) on an ASPEC XL4 liquid handling robot (Gilson). Proteins were collected in elution buffer (8 M urea, 0.1 M NaCl, 29.6 mM HAc, 70.4 mM NaAc, and 50 mM NaH_2_PO_4_, pH 5.0). A Vivaspin concentrator (Sartorius Stedim) was applied to buffer exchange into HBS-EP buffer (5 mM HEPES, 150 mM NaCl, 3.4 mM EDTA, 0.005% p20, pH 7.4).

### Protein analysis

The purity and concentration of purified proteins were examined by SDS-PAGE and bicinchonic acid assay (BCA, Thermo Fisher Scientific/Pierce), respectively. 15% polyacrylamide gels (Criterion ™ Precast Gel, Tris-HCl, Bio Rad Laboratories) was used under reducing conditions and stained with GelCode® Blue Stain (Thermo Fisher Scientific) according to the suppliers recommendations. A LMW-SDS Marker (GE Healthcare) was used as molecular weight marker.

### Surface plasmon resonance (SPR) analyses

In order to analyze the binding characteristics of the mutant domains, two formats were used in a BIAcore 2000 biosensor instrument (Biacore). In a first format, three mouse IgG_1_ mAbs (20 µg/ml solutions of mAb107, HDL110, 7-B6-1, Mabtech, Sweden) were separately immobilized onto discrete CM-5 sensor chip surfaces via amine coupling chemistry to approximately 5000 RU. All mutant domains were separately injected at a concentration of 2 µM and the responses recorded. 10 mM HCl solution was used as regeneration buffer and HBS-EP as running buffer. Some variants were serially diluted to concentrations of 250 nM, 500 nM, 1 µM, 2 µM, 4 µM, 8 µM, 16 µM, 32 µM and 64 µM for affinity measurements. In a second format, a limited number of Z domain variants were immobilized on discrete CM-5 sensor chip surfaces using amine coupling chemistry, followed by injection of 100 nM solutions of different immunoglobulin samples, including four different mIgG_1_ mAbs (mAb107, HDL110, 1D1K and 7-B6-1, Mabtech) and etanercept (Enbrel®).

### Maleimide benzophenone modification

3 mg 4-(N-maleimido) benzophenone (MBP, Sigma-Aldrich) was dissolved in 300 µl dimethylformamide (DMF) (Carlo Erba) to a final concentration of 100 µM. Four µl of 0.5 M tris(2-carboxyethyl)phosphine hydrochloride solution (TCEP) (Sigma-Aldrich, US) was added to 500 µl cysteine-Z_F5I_ variants in PBS buffer (250 µM) and incubated at 37°C (30 min) and then buffer exchanged via PD-5 desalting columns (GE Healthcare) into 50 mM pH 5.5 NaAc buffer. Two µl of the 100 µM MBP solution was immediately added and the tube was covered with foil film in the dark for about 45 mins at 37°C with over-end-over rotation. Subsequently, PD-5 desalting columns were applied to buffer exchange back to PBS buffer. An Accurate-Mass Q-TOF LC/MS 6520 ESI-MS instrument (Agilent Technologies) was used according to the manufacturer's recommendations to assess molecular mass.

### Photo-conjugation of mouse IgG_1_

A 1 mg/ml solution of the mIgG_1_ mAb HDL110 (Mabtech) was diluted in PBS to a final concentration of 1.3 µM concentration as aliquots mixed with each MBP-modified Z domain variant at approximately 6.5 µM (96-well microplate). Each mixture was first incubated at room temperature (30 min) in the dark and then exposed to 365 nm UV light under a Spectroline BLE-8T lamp (Spectronics Corporation) for 2 h on ice in an Ultraviolet Crosslinker Device (Amersham). Photo-conjugation efficiencies were determined through scanning of gel images using ImageJ software (National Institutes of Health, USA). A value of the efficiency (%) for a particular probe was calculated based on the amount of probe-containing Fc fragments divided by the sum of amounts of both probe containing and non-probe containing Fc fragments. For the mean values (Fig. 6B), photo-conjugation efficiencies of probes to 16 of the 19 mAbs shown in Fig. 5B were used (mAbs no. 2, 7 and 12 were excluded due to difficulties in the scanning to fully resolve the different bands).

### Biotinylation

As instructed by the manufacturer, 2.0 mg EZ-link® Sulfo-NHS-LC-Biotin (Thermo Fisher Scientific, USA) was dissolved in 360 µl MilliQ water to obtain a 10 mM solution. To biotinylate the anti-human interferon- gamma mIgG_1_ mAb 1-D1K directly, a 1∶20 molar ratio between the antibody and the Sulfo-NHS-LC-Biotin reagent was incubated at room temperature (30 min) and then transferred into a Slide-A-Lyzer dialysis cassette, 2K MWCO (Thermo Fisher Scientific). The cassette was placed in PBS, pH 7.4 overnight at 4°C. The molar ratio used for biotinylation of the probe Z_F5I-Q32-MBP_ was either 1∶1, 1∶2, 1∶5 or 1∶20. The Z_F5I-Q32-MBP_ sample was first buffer exchanged from 100 mM PBS pH 7.4 into 50 mM PBS, pH 6.5 by using a NAP-10 column (GE Healthcare). The Sulfo-NHS-LC-Biotin reagent was added to the Z_F5I-Q32-MBP_ sample and incubated for 30 min followed by loading onto a NAP-10 column to remove excess biotin.

### Photo coupling between mIgG_1_ mAb 1-D1K and biotinylated Z_F5I-Q32C-MBP_

mIgG_1_ mAb 1-D1K (1 mg/ml) was mixed with a four-fold molar excess of biotinylated Z_F5I-Q32C-MBP_ and incubated at room temperature (1 hr) in the dark followed by 2 hr exposure to UV light as described above. After photocoupling, non-covalently bound biotinylated Z_F5I-Q32C-MBP_ probe was removed with a 30 kDa cutoff Vivaspin 500 column (Sartorius Stedim) after adjustment of the sample pH to 3.3 with 0.3 mM HAc buffer. Finally, the buffer was exchanged again to PBS pH 7.4.

### Western Blotting

About 3 µg of either biotinylated mAb 1-D1K (standard procedure), biotinylated Z_F5I-Q32C-MBP_ probe or probe-biotinylated mAb 1-D1K was subjected to SDS-PAGE separation under reducing conditions using a NuPAGE® Novex 4–12% Bis-Tris Gel (Invitrogen). Proteins were transferred to a PVDF membrane and biotinylated species visualized using a Streptavidin-HRP conjugate (DakoCytomation, Denmark).

### Bioactivity of an biotinylated anti-human interferon-gamma mouse IgG_1_ mAb

A streptavidin coated SA BIAcore sensor chip (GE Healthcare, Sweden) was cleaned with three 1 min injections of 1M NaCl in 50 mM NaOH before immobilization of ligands. 1.5 µg/ml solutions of either the probe-biotinylated mAb 1-D1K or the conventionally biotinylated mAb 1-D1K were repeatedly injected over separate surfaces to effect immobilization (final response levels were 1596 and 1944 RU, respectively). After immobilization of a biotinylated sample, the microfluidics channels were subjected an extra wash with 50% isopropanol in 1 M NaCl and 50 mM NaOH to clean the needle and IFC sample line from biotinylated material. A 7.5 nM solution of human interferon gamma (R&D systems, UK) in PBS-T (0.005%) buffer was injected over the sensor chip at a flow rate of 20 µl/min for 4 min.

## Supporting Information

Experiment S1 **Mapping of the conjugation site on mIgG_1_.** The photo-conjugation site for the biotinylated Z_F5I-Q32C-MBP_ probe on the heavy chain was mapped to the Fc fragment, and not to the domains of the heavy chain that are part of the F(ab´)_2_ fragment.
(DOC)

## References

[1] Tavoosidana G, Ronquist G, Darmanis S, Yan J, Carlsson L, et al (2011) Multiple recognition assay reveals prostasomes as promising plasma biomarkers for prostate cancer. Proc Natl Acad Sci U S A 108: 8809–8814. 21555566 10.1073/pnas.1019330108 PMC3102389

[2] Stadler C, Hjelmare M, Neumann B, Jonasson K, Pepperkok R, et al (2012) Systematic validation of antibody binding and protein subcellular localization using siRNA and confocal microscopy. J Proteomics 75: 2236–2251. 22361696 10.1016/j.jprot.2012.01.030

[3] Gallagher S, Winston SE, Fuller SA, Hurrell JG (2011) Immunoblotting and immunodetection. Curr Protoc Cell Biol Chapter 6: Unit6 2 10.1002/0471143030.cb0602s52 21898339

[4] Clausson CM, Allalou A, Weibrecht I, Mahmoudi S, Farnebo M, et al (2011) Increasing the dynamic range of in situ PLA. Nat Methods 8: 892–893. 22036742 10.1038/nmeth.1743

[5] Vashist SK (2012) Effect of antibody modifications on its biomolecular binding as determined by surface plasmon resonance. Anal Biochem 421: 336–338. 22093612 10.1016/j.ab.2011.10.036

[6] Konrad A, Karlstrom AE, Hober S (2011) Covalent immunoglobulin labeling through a photoactivable synthetic Z domain. Bioconjug Chem 22: 2395–2403. 22026370 10.1021/bc200052h

[7] Jung Y, Lee JM, Kim JW, Yoon J, Cho H, et al (2009) Photoactivable antibody binding protein: site-selective and covalent coupling of antibody. Anal Chem 81: 936–942. 19133771 10.1021/ac8014565

[8] Holm L, Moody P, Howarth M (2009) Electrophilic affibodies forming covalent bonds to protein targets. J Biol Chem 284: 32906–32913. 19759009 10.1074/jbc.M109.034322 PMC2781706

[9] Nilsson B, Moks T, Jansson B, Abrahmsen L, Elmblad A, et al (1987) A synthetic IgG-binding domain based on staphylococcal protein A. Protein Eng 1: 107–113. 3507693 10.1093/protein/1.2.107

[10] Nagaoka M, Akaike T (2003) Single amino acid substitution in the mouse IgG1 Fc region induces drastic enhancement of the affinity to protein A. Protein Eng 16: 243–245. 12736366 10.1093/proeng/gzg037

[11] Page M, Thorpe R (1998) Purification of monoclonal antibodies. Methods Mol Biol 80: 113–119. 9664368 10.1007/978-1-59259-257-9_11

[12] Deisenhofer J (1981) Crystallographic refinement and atomic models of a human Fc fragment and its complex with fragment B of protein A from Staphylococcus aureus at 2.9- and 2.8-A resolution. Biochemistry 20: 2361–2370. 7236608

[13] Kato K, Gouda H, Takaha W, Yoshino A, Matsunaga C, et al (1993) 13C NMR study of the mode of interaction in solution of the B fragment of staphylococcal protein A and the Fc fragments of mouse immunoglobulin G. FEBS Lett 328: 49–54. 8344434 10.1016/0014-5793(93)80963-u

[14] Chapman-Smith A, Cronan JE Jr (1999) In vivo enzymatic protein biotinylation. Biomol Eng 16: 119–125. 10796994 10.1016/s1050-3862(99)00046-7

[15] Schatz PJ (1993) Use of peptide libraries to map the substrate specificity of a peptide-modifying enzyme: a 13 residue consensus peptide specifies biotinylation in Escherichia coli. Biotechnology (N Y) 11: 1138–1143. 7764094 10.1038/nbt1093-1138

[16] Beckett D, Kovaleva E, Schatz PJ (1999) A minimal peptide substrate in biotin holoenzyme synthetase-catalyzed biotinylation. Protein Sci 8: 921–929. 10211839 10.1110/ps.8.4.921 PMC2144313

