# Supplementary material for: Tailor-Making a Protein A-Derived Domain for Efficient Site-Specific Photocoupling to Fc of Mouse IgG1
Source: PLoS One. 2013 Feb 12;8(2):e56597. doi: 10.1371/journal.pone.0056597 (PMC3570467; doi:10.1371/journal.pone.0056597)
Supplement: Experiment S1 — Mapping of the conjugation site on mIgG1. The photo-conjugation site for the biotinylated ZF5I-Q32C-MBP probe on the heavy chain was mapped to the Fc fragment, and not to the domains of the heavy chain that are part of the F(ab´)2 fragment. (DOC) [file pone.0056597.s001.doc]

**SUPPORTIVE INFORMATION**

**Tailor-making a protein A-derived domain for efficient site-specific photocoupling to Fc of mouse IgG_1_**

**Short title: Fc-specific photoconjugation to mouse IgG_1_**

Feifan Yu^1^, Peter Järver^1,2^ and Per-Åke Nygren^1,*^

(1) Division of Molecular Biotechnology
Royal Institute of Technology (KTH)
AlbaNova University Center
Stockholm
Sweden

(2) *Present address:*MRC Laboratory of Molecular Biology
PNAC Division
Cambridge
United Kingdom

*(*) Corresponding author*
E-mail: perake@biotech.kth.se
Tel: +46-8-553783

Experiment S1. Mapping of the conjugation site on mIgG_1_.

The SDS-PAGE analysis of the coupling efficiency (Fig. 5) revealed that only heavy chains had been coupled and that no unspecfic coupling was seen to the light chains of the mAbs. However, the analysis could not show if it was the Fc fragment or the VH-CH_1_ portion of the heavy chain had been coupled to the probe. To investigate this further, a mapping experiment was performed. To this end, mAb 1-D1K protein biotinylated using the bio-Z_F5I-Q32C-MBP_ probe was first digested with papain, cleaving the antibody into Fc and F(ab´)_2_ fragments. To investigate what parts of the digested antibody that contained biotin, and thus had been coupled to the bio-Z_F5I-Q32C-MBP_ probe, the cleavage mixture was incubated with streptavidin coated microbeads (SA-beads) for capture of any biotinylated protein. The supernatant from this incubation was saved. In a following dot blot analysis, samples corresponding to material eluted from the SA-beads and the supernatant from the bead incubation were analyzed using anti-mouse IgG Fab and anti-mouse IgG Fc immunoconjugates, respectively.

The results showed that the eluate from the SA-beads was recognized by the anti-mouse IgG Fc reagent but not by the anti-mouse IgG Fab reagent (Fig. S1, a and b). The supernatant sample was recognized by both the anti-mouse IgG Fc (weakly) and the anti-mouse IgG Fab reagents (Fig. S1, b and c). Taken together, this indicates that the biotinylation of the mAb 1-D1K using the bio-Z_F5I-Q32C-MBP_ probe had only occured on the Fc fragment and that the Fab portion had not been biotinylated and thus remained in the supernatant after the incubation of the cleavage mixture with the SA-beads. The signal from using anti-mouse IgG Fc reagent on the supernatant sample indicates that a portion of the mAb had not been biotinylated or that the capacity of the SA-beads had been exceeded.


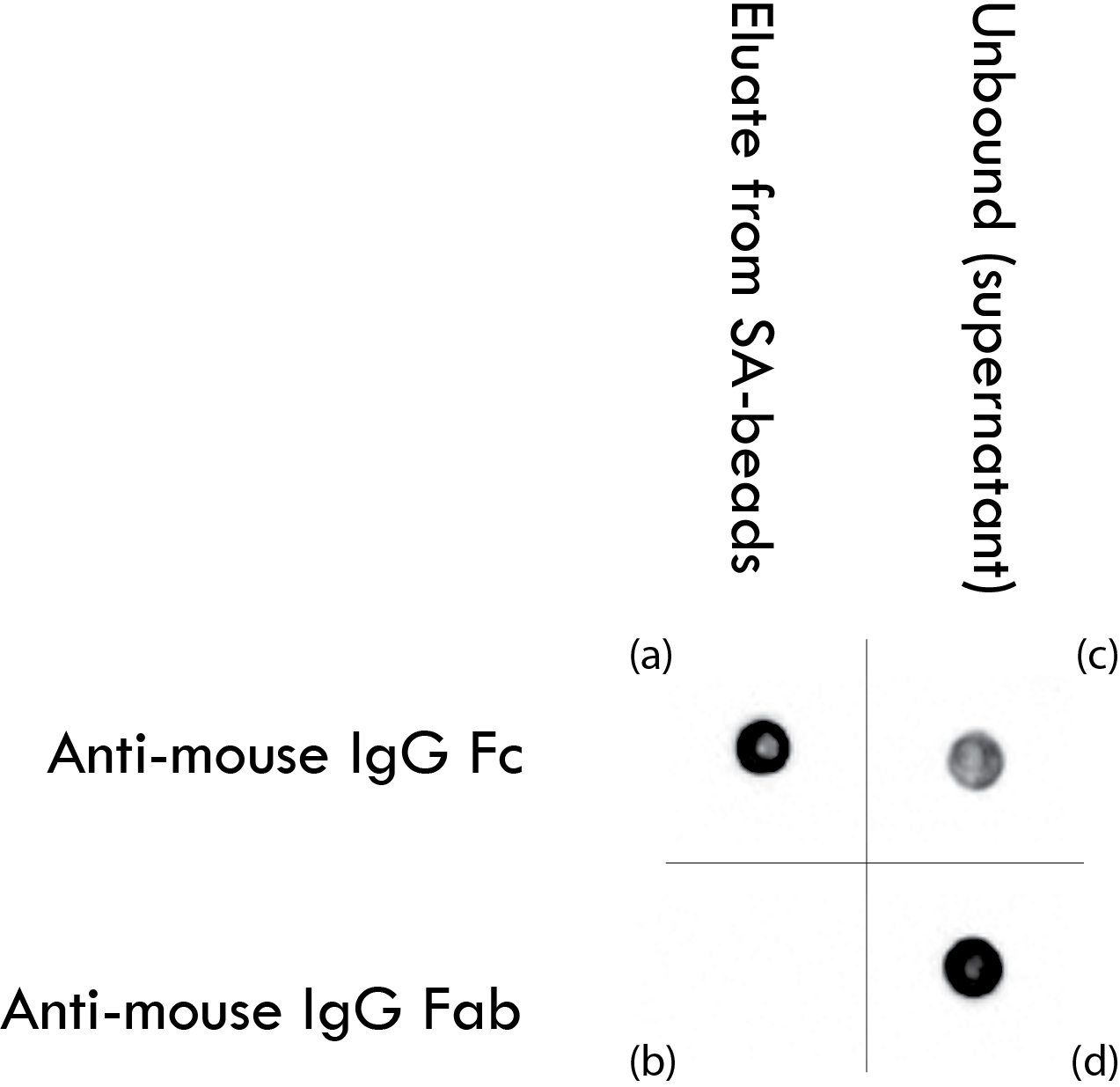


**Figure S1. Dot blot analysis.** Results from a dot blot analysis designed to determine what region(s) of the heavy chain of a full-sized monoclonal mouse IgG_1_ antibody (mAb) that had become photo-coupled to a biotinylated Z_F5I-Q32C-MBP_ probe. Papain digestion was used to produce Fc and F(ab´)_2_ fragments of the biotinylated mAb. Streptavidin coated beads were subsequently used for capture of any biotinylated proteins, the identity of which was analysed in a dot blot experiment using anti-mouse IgG-Fc and anti-mouse IgG F(ab’)_2_ immunoreagents, respectively. The results showed that only Fc fragments had been captured on the SA-beads (panels a and b), and that the F(ab’)_2_ fragments (and a small amount of Fc fragments) had remained in the supernatant during SA-bead incubation. This indicates a selective photo-coupling to the Fc fragment of the mAb. See text for details.

Methods

20 μg of mAb 1-D1K biotinylated with the Bio-Z_F5I-Q32C-MBP_ probe was buffer exchanged into papain digestion buffer (20 mM sodium phosphate, 10 mM EDTA and 20 mM L-Cysteine, pH 7.0) using a PD SpinTrap G25 column (GE Healthcare, Sweden). 100 μl of beads with immobilized papain (Thermo Fisher Scientific/Pierce) were washed by digestion buffer twice and mixed with antibody. The cleavage reaction was carried on at 37°C over night with rotation. After centrifuging at 1300 *g* for 1 min, the supernatant of the digestion mixture was separated and incubated with Dynabeads® M280 Streptavidin beads (Invitrogen) (SA-beads) for 30 minutes at room temperature with rotation. After the incubation the supernatant was saved (supernatant sample) and the SA-beads washed. Proteins captured on SA-beads were removed by heating the beads. Samples from the boiled SA-beads and the supernatant, respectively, were analyzed by dot blotting. Five microlitre-samples were spotted on separate 0.45 μM nitrocellulose membrane pieces (Invitrogen) followed by incubation with a goat F(ab’)_2_ polyclonal antibody against mouse IgG-Fc HRP (ab5879, Abcam) (1:1000 dilution) and goat F(ab’)_2_ polyclonal antibody against IgG-F(ab’)_2_ HRP (ab5887, Abcam) (1:2000 dilution), respectively. The membranes were developed using standard HRP substrate reagents for enhanced chemiluminescence analysis.
